# Clinical characteristics of different subtypes and risk factors for the severity of illness in patients with COVID-19 in Zhejiang, China

**DOI:** 10.1186/s40249-020-00710-6

**Published:** 2020-07-08

**Authors:** Shan-Yan Zhang, Jiang-Shan Lian, Jian-Hua Hu, Xiao-Li Zhang, Ying-Feng Lu, Huan Cai, Jue-Qing Gu, Chan-Yuan Ye, Ci-Liang Jin, Guo-Dong Yu, Hong-Yu Jia, Yi-Min Zhang, Ji-Fang Sheng, Lan-Juan Li, Yi-Da Yang

**Affiliations:** State Key Laboratory for Diagnosis and Treatment of Infectious Diseases, National Clinical Research Center for Infectious Diseases, Collaborative Innovation Center for Diagnosis and Treatment of Infectious Diseases, The First Affiliated Hospital, College of Medicine, Zhejiang University, Hangzhou, China

**Keywords:** COVID-19, SARS-CoV-2, Subtype, Risk factor, Gastrointestinal symptom

## Abstract

**Background:**

The outbreak of coronavirus disease 2019 (COVID-19) is now becoming an enormous threat to public health. The clinical spectrum of COVID-19 is extensive, of which critical cases are with rapid disease progression and high mortality. The aim of our study is to summarize the characteristics of different subtypes and explore risk factors of illness severity for early identification and prompt treatment.

**Methods:**

In this retrospective study, we collected data of patients confirmed COVID-19 in Zhejiang Province from 17 January to 12 February 2020. According to the definition of clinical classification, we divided confirmed cases into four types, and summarize epidemiological and clinical characteristics, laboratory and radiograph findings, treatments, and outcomes, respectively. Moreover, we used univariate and multivariate ordinal logistic regression models to explore risk factors for the severity of illness in patients with COVID-19.

**Results:**

A total of 788 patients were enrolled in our study, of whom 52 cases (6.6%) were mild type, 658 cases (83.5%) were common type, 61 cases (7.2%) were severe type, and 17 cases (2.2%) were critical type. Multivariate ordinal logistic regression demonstrated increasing odds of the severity of illness in patients with COVID-19 associated with male (odds ratio [*OR*] = 1.7, 95% confidence interval [*CI*]: 1.2–2.6 *P* = 0.008), fever (*OR* = 3.6, 95% *CI*: 2.1–6.3, *P* <  0.001), cough (*OR* = 1.7, 95% *CI*: 1.0–2.9, *P* = 0.041), hemoptysis (*OR* = 3.4, 95% *CI*: 1.1–10.3, *P* = 0.032), gastrointestinal symptoms (*OR* = 1.9, 95% *CI*: 1.0–3.5, *P* = 0.047), hypertension (*OR* = 2.6, 95% *CI*: 1.2–5.6, *P* = 0.013). With the increase of age-grading, risk for the severity of illness was gradually higher (≤ 18 years [*OR* = 1.0], 19–40 years [*OR* = 12.7, 95% *CI*: 4.5–36.0, *P* < 0.001], 41–65 years [*OR* = 14.8, 95% *CI*: 5.2–42.1, *P* <  0.001], ≥ 66 years [*OR* = 56.5, 95% *CI*: 17.1–186.5, *P* < 0.001]).

**Conclusions:**

Clinicians should pay close attention to these features in patients with COVID-19 including older age, male, fever, cough, hemoptysis, gastrointestinal symptoms and hypertension to identify the severity of illness as early as possible.

## Background

In December, 2019, a cluster of patients with pneumonia of unknown cause appeared in Wuhan, Hubei Province, China. By 7 January 2020, China rapidly isolated the novel coronavirus and shared the viral genome sequence to World Health Organization (WHO) [1]. The novel coronavirus was identified as a novel enveloped RNA betacoronavirus and named 2019 novel coronavirus (later named as severe acute respiratory syndrome coronavirus 2 [SARS-CoV-2] by the International Committee on Taxonomy of Viruses), which has a phylogenetic similarity to severe acute respiratory syndrome coronavirus (SARS-CoV), but the contagosity is higher than SARS-CoV and Middle East respiratory syndrome coronavirus (MERS-CoV) [2]. In order to prevent the epidemic of this contagion, Chinese government made a decision to temporarily shut down the traffic departing from Wuhan on 23 January 2020 and adopted a series of control measures.

WHO declared the spread of SARS-CoV-2 was listed as a public health emergency of international concern, and subsequently designated the pneumonia infected by SARS-CoV-2 as coronavirus disease 2019 (COVID-19) [3]. Until 19 April 2020, COVID-19 has swept across 213 countries and regions which reported 2 245 872 confirmed cases and 152 707 deaths. The largest number of confirmed cases were in the United States, followed by Spain and Italy [4]. This phenomenon signified fighting with COVID-19 is not only a matter for China, but an imperative event for global.

Nowadays, the majority of studies on COVID-19 in China are focused on Wuhan, the hardest-hit area, and little is known about the clinical features of COVID-19 outside of Wuhan [5–7]. The study of Chang et al. [8] included 13 cases in Beijing and the research of Xu et al. [9] enrolled 62 cases in Zhejiang, however, due to the small sample size, clinical characteristics might be not comprehensive. Researches with a larger number of confirmed cases were urgently needed outside of Wuhan. The clinical spectrum of COVID-19 appears to be wide, comprising mild type without pneumonia, common type with pneumonia, severe type with respiratory distress, and critical type with respiratory failure, shock or even death [10]. Diverse subtypes have their unique features, whether in epidemiology or laboratory results. A study with 72 314 cases reported by the Chinese Center for Disease Control and Prevention (China CDC) showed that the case-fatality rate was 49.0% among critical cases [11]. Therefore, mastering the characteristics of different subtypes and early identification of the severity of illness is of great significance for the treatment.

Hence, the aim of our study is to summarize the epidemiologic and clinical characteristics, laboratory and radiograph findings, treatments, and outcomes of different subtypes of patients with COVID-19 in Zhejiang Province. On this basis, we want to explore risk factors for the severity of illness in patients with COVID-19 and appeal to clinicians to attach importance to these factors.

## Methods

### Data sources and ethics

We conducted a retrospective study investigating on the epidemiological, clinical, laboratory, radiograph, treatments and outcomes characteristics of confirmed cases of COVID-19 in Zhejiang Province from 17 January to 12 February 2020. Diagnosis of COVID-19 was in accordance with the interim guidance from the WHO [12]. A confirmed case of COVID-19 was defined as a positive result on real-time reverse transcriptase polymerase chain reaction (RT-PCR) assay of sputum and throat swab specimens. Only laboratory-confirmed patients were enrolled in our study. Data were uniformly collected by the Health Commission of Zhejiang Province, where all patients were allocated at specific hospitals for unified treatment according to the government emergency rule. All data enrolled in our study had been shared with WHO and the preliminary results were reported to the authority of Zhejiang Province.

Information of medical records were gathered and sent to the data collection center in Hangzhou. Demographic, epidemiological, clinical, laboratory, treatments and outcomes data were exacted from electronic medical records using a standardized data collection form. A group of doctors who have experiences in treating the patients with COVID-19 reviewed and disposed the data. When information was incomplete, the working team in Hangzhou would contact the doctor in charge for explanation.

This study was approved by the Clinical Research Ethics Committee of the First Affiliated Hospital, College of Medicine, Zhejiang University (No. IIT20200005C). Written informed consent was waived by the ethics commission of the designated hospital, and oral consent was obtained from patients.

### Procedures

Sputum and throat swab specimens collected from all patients were tested by RT-PCR for SARS-CoV-2 RNA. Briefly, the CDC of Zhejiang Province and at municipal level, and the First Affiliated Hospital, School of Medicine, Zhejiang University were responsible for confirmation of SARS-CoV-2, with national authorization.

Laboratory tests were conducted on admission, including blood routine examinations, serum biochemical tests, coagulation function examinations, infection-related biomarkers, and an identification of other respiratory pathogens such as influenza A virus, influenza B virus, parainfluenza virus, adenovirus and respiratory syncytial virus. Chest radiograph or computed tomography (CT) was done for all inpatients at admission. Treatment measures and outcomes were followed up to 12 February 2020.

### Case definitions

The illness severity of COVID-19 was defined according to the Chinese management guideline for COVID-19 (version 6.0) [13]. Patients with COVID-19 was categorized as mild, common, severe, and critical according to the illness severity. Mild type was defined as mild symptoms and no pneumonia on imaging. Common type was defined as having respiratory tract symptoms and imaging with pneumonia. Severe type was defined as satisfying any of the following items: 1) respiratory distress and respiratory frequency ≥ 30/min; 2) blood oxygen saturation ≤ 93% at rest; 3) PaO_2_/FiO_2_ ratio ≤ 300 mmHg; 4) Lung infiltrates > 50% within 24–48 h. Critical type was defined as satisfying any of the following items: 1) respiratory failure occurs and require mechanical ventilation; 2) shock occurs; 3) combined with other organ failure and requires ICU monitoring and treatment. The incubation period was defined as the time from exposure to the onset of illness, which was estimated among patients who could provide the exact data of close contact with confirmed or suspected individuals. Family cluster was defined as occurring two or more cases with fever and/or respiratory symptoms within the family in recent 2 weeks. Fever was defined as axillary temperature of at least 37.3 °C. Gastrointestinal symptoms included nausea, emesis and diarrhea.

### Discharge criteria

Once the temperature returned to normal for more than 3 days, respiratory symptoms significantly improved, the imaging of lung obviously absorbed, and two consecutive negative results for SARS-CoV-2 antigen (sampling interval at least 1 day), patients could be discharged from hospital.

### Statistical analysis

Continuous variables were presented as median (interquartile range [IQR]) and compared using Kruskal-Wallis. Categorical variables were presented as frequency (percentages), and compared using the *χ*^2^ test or Fisher’s exact test when appropriate.

To analyze risk factors for the severity of illness in patients with COVID-19, univariate and multivariate ordinal logistic regression models were used. Variables with *P <* 0.05 in the univariate models were selected into the multivariate model for calculating. A two-sided α of *<* 0.05 was considered statistically significant. All the analyses were done with SPSS version 25.0 (IBM Corporation, Armonk, NY, USA).

The Kaplan-Meier method was used to estimate hospitalization time, and the log rank test was applied for comparisons among mild, common, and severe type. The Kaplan-Meier analysis was performed using ‘survival’ packages in R version 3.6.1 (R Foundation, Vienna, Austria).

## Results

### Demographic, epidemiologic, and clinical characteristics

From 17 January 2020 to 12 February 2020, clinical data were collected on 788 patients with COVID-19 in Zhejiang Province. According to the definition of clinical classification, they were divided into 52 cases (6.6%) of mild type, 658 cases (83.5%) of common type, 61 cases (7.7%) of severe type, and 17 cases (2.2%) of critical type.

As shown in Table 1, the median age in mild, common, severe, and critical type was 37.5 years (IQR: 19.3–45.8), 45.0 years (IQR: 35.0–55.0), 55.0 years (IQR: 44.0–62.0), and 70.0 years (IQR: 55.0–73.0). The proportion of male patients was account for 50.0, 50.0, 63.9 and 76.5%, respectively. Hypertension was the most common underlying disease and there were significant differences among the four subtypes (9.6% vs 13.2% vs 31.1% vs 88.2%, *P* < 0.001). In both severe and critical type, the ratio of patients with gastrointestinal symptoms exceeded 15%.

**Table 1:** Demographic, epidemiologic and clinical characteristics of different subtypes in patients with COVID-19

**Characteristic**	**Mild type (*n*** **= 52)**	**Common type (*n*** **= 658)**	**Severe type (*n*** **= 61)**	**Critical type (*n*** **= 17)**	***P*value**
**Age** (years)	37.5 (19.3–45.8)	45.0 (35.0–55.0)	55.0 (44.0–62.0)	70.0 (55.0–73.0)	**< 0.001**
Distribution					**< 0.001**
≤ 18	12 (23.1)	9 (1.4)	0 (0.0)	0 (0.0)	
19–40	16 (30.8)	241 (36.6)	11 (18.0)	0 (0.0)	
41–65	24 (46.2)	350 (53.2)	39 (63.9)	7 (41.2)	
≥ 66	0 (0.0)	58 (8.8)	11 (18.0)	10 (58.8)	
**Sex (male)**	26 (50.0)	329 (50.0)	39 (63.9)	13 (76.5)	**0.034**
**BMI** (kg/m^2^)					**< 0.001**
< 18.5	5/25 (20.0)	20/370 (5.4)	0/36 (0.0)	0/13 (0.0)	
18.5–< 25	17/25 (68.0)	240/370 (64.9)	17/36 (47.2)	7/13 (53.8)	
≥ 25	3/25 (12.0)	110/370 (29.7)	19/36 (52.8)	6/13 (46.2)	
**Current smoker**	2 (3.8)	45 (6.8)	5 (8.2)	2 (11.8)	0.544
**Exposure history in Wuhan**	23 (44.2)	331 (50.3)	31 (50.8)	8 (47.1)	0.853
**Incubation period** (days)	7.0 (3.0–11.5) (*n* = 17)	6.0 (3.0–9.0) (*n* = 156)	3.0 (2.0–5.5) (*n* = 14)	7.0 (*n* = 1)	0.239
**Family cluster**	25 (48.1)	152 (23.1)	11 (18.0)	7 (41.2)	**< 0.001**
**Time from illness onset to first hospital admission** (days)	2.5 (1.0–4.0)	3.0 (1.0–6.0)	5.0 (2.0–8.0)	5.0 (2.5–6.5)	**< 0.001**
**Coexisting disorder**					
Any	10 (19.2)	161 (24.5)	31 (50.8)	16 (94.1)	**< 0.001**
Hypertension	5 (9.6)	87 (13.2)	19 (31.1)	15 (88.2)	**< 0.001**
Heart disease	0 (0.0)	9 (1.4)	1 (1.6)	1 (5.9)	0.254
Diabetes	3 (5.8)	42 (6.4)	8 (13.1)	4 (23.5)	**0.019**
Chronic obstructive pulmonary disease	0 (0.0)	0 (0.0)	2 (3.3)	1 (5.9)	**0.001**
Cancer	0 (0.0)	3 (0.5)	3 (4.9)	0 (0.0)	**0.030**
Chronic liver disease	1 (1.9)	26 (4.0)	2 (3.3)	2 (11.8)	0.351
Chronic renal disease	0 (0.0)	5 (0.8)	1 (1.6)	1 (5.9)	0.150
**Symptoms on admission**					
Fever	28 (53.8)	534 (81.2)	57 (93.4)	17 (100.0)	**< 0.001**
Cough	19 (36.5)	426 (64.7)	49 (80.3)	12 (70.6)	**< 0.001**
Sputum production	8 (15.4)	219 (33.3)	29 (47.5)	9 (52.9)	**0.001**
Hemoptysis	0 (0.0)	8 (1.2)	6 (9.8)	1 (5.9)	**0.001**
Sore throat	8 (15.4)	95 (14.4)	7 (11.5)	1 (5.9)	0.796
Nasal obstruction	6 (11.5)	41 (6.2)	0 (0.0)	0 (0.0)	**0.034**
Myalgia	4 (7.7)	68 (10.3)	14 (23.0)	5 (29.4)	**0.004**
Fatigue	10 (19.2)	105 (16.0)	18 (29.5)	6 (35.3)	**0.013**
**Gastrointestinal symptom**	7 (13.5)	63 (9.6)	12 (19.7)	6 (35.3)	**0.002**
Headache	2 (3.8)	61 (9.3)	11 (18.0)	1 (5.9)	0.076

Data are presented as medians (interquartile ranges, IQR), *n* (%) and *n*/*N* (%)

### Laboratory and radiograph findings

On admission, the majority of leucocyte in all subtypes were normal or decreased. As shown in Table 2, the four types had significant differences in neutrophil count, lymphocyte count, platelets count, albumin (ALB), aspartate aminotransferase (AST), sodium, blood urea nitrogen (BUN), creatinine (CR), creatinine kinase (CK), lactate dehydrogenase (LDH), C-reactive protein (CRP) and procalcitonin (PCT) (*P* < 0.05). In contrast, there were no significant differences in hemoglobin, International normalized ration (INR), alanine aminotransferase (ALT), total bilirubin (TB) and potassium (*P* > 0.05).

**Table 2:** Laboratory and radiograph findings of different subtypes in patients with COVID-19 on admission

**Variable**	**Mild type (*n*** **= 52)**	**Common type (*n*** **= 658)**	**Severe type (*n*** **= 61)**	**Critical type (*n*** **= 17)**	***P*value**
**Blood routine**					
Leucocyte count (× 10^9^/L)	5.7 (4.3–7.1)	4.7 (3.8–5.8)	4.9 (3.8–6.6)	6.8 (3.8–8.8)	**0.002**
< 4	9 (17.3)	201 (30.5)	19 (31.1)	5 (29.4)	
> 10	1 (1.9)	10 (1.5)	4 (6.6)	3 (17.6)	
Neutrophil count (× 10^9^/L)	3.6 (2.0–5.0)	2.9 (2.2–3.8)	3.2 (2.6–5.0)	5.8 (2.8–8.0)	**< 0.001**
> 7	3 (5.8)	17 (2.6)	7 (11.5)	7 (41.2)	
Lymphocyte count (× 10^9^/L)	1.3 (1.1–1.9)	1.2 (0.9–1.6)	0.9 (0.6–1.2)	0.5 (0.4–0.8)	**< 0.001**
< 0.8	8 (15.4)	91 (13.8)	23 (37.7)	12 (70.6)	
Hemoglobin (g/L)	139.0 (131.0–152.0)	138.0 (127.0–150.0)	139.0 (122.5–153.0)	128.0 (117.0–153.5)	0.332
Platelets count (×10^9^/L)	206.0 (171.0–241.3)	180.0 (147.8–221.3)	172.0 (138.0–214.0)	146.0 (122.0–181.5)	**0.001**
< 100	0 (0.0)	23 (3.5)	2 (3.3)	2 (11.8)	
**Coagulation function**					
International normalized ration (INR)	1.0 (1.0–1.1)	1.0 (1.0–1.1)	1.0 (1.0–1.1)	1.0 (1.0–1.2)	0.070
**Blood biochemistry**					
Albumin (ALB, g/L)	42.5 (40.5–45.7)	41.7 (38.7–43.9)	38.7 (35.8–41.6)	35.9 (30.8–37.6)	**< 0.001**
Alanine aminotransferase (ALT, U/L)	20.0 (12.0–39.1)	21.1 (15.0–33.0)	24.0 (16.5–34.5)	20.5 (14.0–30.8)	0.528
Aspartate aminotransferase (AST, U/L)	22.0 (16.5–34.0)	25.0 (19.1–32.2)	28.0 (22.0–40.0)	30.5 (25.0–41.8)	**0.002**
> 40	7 (13.5)	76 (11.6)	13 (21.3)	5 (29.4)	
Total bilirubin (TB) (μmol//L)	8.8 (6.2–11.6)	9.5 (7.0–13.1)	10.7 (7.8–15.6)	10.3 (8.0–14.6)	0.205
Potassium (mmol/L)	3.8 (3.6–4.2)	3.8 (3.6–4.1)	3.9 (3.6–4.2)	3.7 (3.2–3.9)	0.112
Sodium (mmol/L)	139.0 (138.0–141.0)	138.5 (136.3–140.2)	137.4 (135.0–139.3)	136.0 (130.1–137.8)	**< 0.001**
Blood urea nitrogen (BUN, mmol/L)	4.2 (3.2–4.7)	3.7 (3.0–4.6)	4.0 (3.2–5.6)	5.8 (3.6–12.3)	**0.001**
Creatinine (CR, μmol/L)	66.0 (58.0–76.0)	65.3 (55.0–78.0)	71.0 (62.5–80.5)	79.0 (65.9–106.5)	**0.002**
Creatinine kinase (CK, U/L)	60.5 (42.5–75.3)	68.5 (47.0–105.3)	76.0 (56.5–120.5)	146.0 (54.3–255.5)	**< 0.001**
Lactate dehydrogenase (LDH, U/L)	175.0 (147.0–241.0)	207.5 (168.0–254.0)	272.0 (221.5–366.5)	320.5 (256.3–356.5)	**< 0.001**
> 250	11 (21.2)	167 (25.4)	39 (63.9)	13 (76.5)	
**Infection-related biomarkers**					
C-reactive protein (CRP, mg/L)	1.9 (0.5–5.5)	7.7 (2.5–19.4)	26.2 (10.4–51.1)	45.5 (14.7–84.9)	**< 0.001**
Procalcitonin (PCT, ng/ml)	0.1 (0.0–0.1)	0.1 (0.0–0.1)	0.1 (0.0–0.1)	0.1 (0.0–0.2)	**0.013**
**Chest x-ray/CT finding**					
Multiple mottling and ground-glass opacity	0(0.0)	183(27.8)	37 (60.7)	15 (88.2)	**< 0.001**

Data are presented as medians (interquartile ranges, IQR), *n* (%) and *n*/*N* (%)

Multiple mottling and ground-glass opacity were typical imaging manifestations of patients with COVID-19. The proportion of it in common, severe, and critical type were 27.8, 60.7, and 88.2%, respectively.

### Treatments and outcomes

Patients with COVID-19 were quarantined in designated hospital and a total of 668 patients received antiviral treatments in our research. As shown in Table 3, interferon-α inhalation, lopinavir/ritonavir and arbidol were the most commonplace antiviral regimen in all subtypes. Glucocorticoids were not used in mild type, but in all critical types, and there were significant differences in the use among the four types (0.0% vs 8.2% vs 47.5% vs 100.0%, *P* <  0.001). Similar to glucocorticoids, there were significant differences among the four types based on the use intravenous immunoglobulin (0.0% vs 3.6% vs 42.6% vs 70.6%, *P* <  0.001). Till 12 February 2020, none of patients used extracorporeal membrane oxygenation (ECMO) and continuous renal-replacement therapy (CRRT).

**Table 3:** Treatments, and clinical outcomes of different subtypes in patients with COVID-19

**Variable**	**Mild type (*n*** **= 52)**	**Common type (*n*** **= 658)**	**Severe type (*n*** **= 61)**	**Critical type (*n*** **= 17)**	***P*value**
Shock	0 (0.0)	0 (0.0)	0 (0.0)	2 (11.8)	**< 0.001**
**Time from illness onset to antiviral treatments** (days)	3.0 (1.0–4.5)	4.0 (2.0–7.0)	7.0 (4.0–9.0)	5.0 (2.0–6.8)	**< 0.001**
Antiviral treatments	41 (78.8)	552 (83.9)	59 (98.7)	16 (94.1)	**0.010**
Interferon-α inhalation + lopinavir/ritonavir	9/41 (22.0)	139/552 (25.2)	16/59 (27.1)	1/16 (6.3)	
Interferon-α inhalation+ arbidol	1/41 (2.4)	33/552 (6.0)	7/59 (11.9)	0/16 (0.0)	
Interferon-α inhalation + lopinavir/ritonavir+ arbidol	14/41 (34.1)	191/552 (34.6)	23/59 (39.0)	7/16 (43.8)	
Lopinavir/ritonavir + arbidol	6/41 (14.6)	62/552 (11.2)	1/59 (1.7)	4/16 (25.0)	
Lopinavir/ritonavir	5/41 (12.2)	60/552 (10.9)	3/59 (5.1)	0/16 (0.0)	
Arbidol	2/41 (4.9)	33/552 (6.0)	6/61 (10.2)	0/16 (0.0)	
Interferon-α inhalation only	4/41 (9.8)	29/552 (5.3)	2/61 (3.4)	0/16 (0.0)	
others	0/41 (0.0)	5/552 (0.9)	1/61 (1.7)	4/16 (25.0)	
**Glucocorticoids**	0 (0.0)	54 (8.2)	29 (47.5)	17 (100.0)	**< 0.001**
**Intravenous immunoglobulin**	0 (0.0)	24 (3.6)	26 (42.6)	12 (70.6)	**< 0.001**
**Extracorporeal membrane oxygenation (ECMO)**	0 (0.0)	0 (0.0)	0 (0.0)	0 (0.0)	
**Continuous renal-replacement therapy (CRRT)**	0 (0.0)	0 (0.0)	0 (0.0)	0 (0.0)	
**Clinical outcomes at data cutoff**					
Discharge from hospital	21 (40.4)	273 (41.5)	27 (44.3)	1 (5.9)	0.029
Hospitalization	31 (59.6)	385 (58.5)	34 (55.7)	16 (94.1)	0.029
Death	0 (0.0)	0 (0.0)	0 (0.0)	0 (0.0)	

Data are presented as medians (interquartile ranges, IQR), *n* (%) and *n*/*N* (%)

**Fig. 1:**
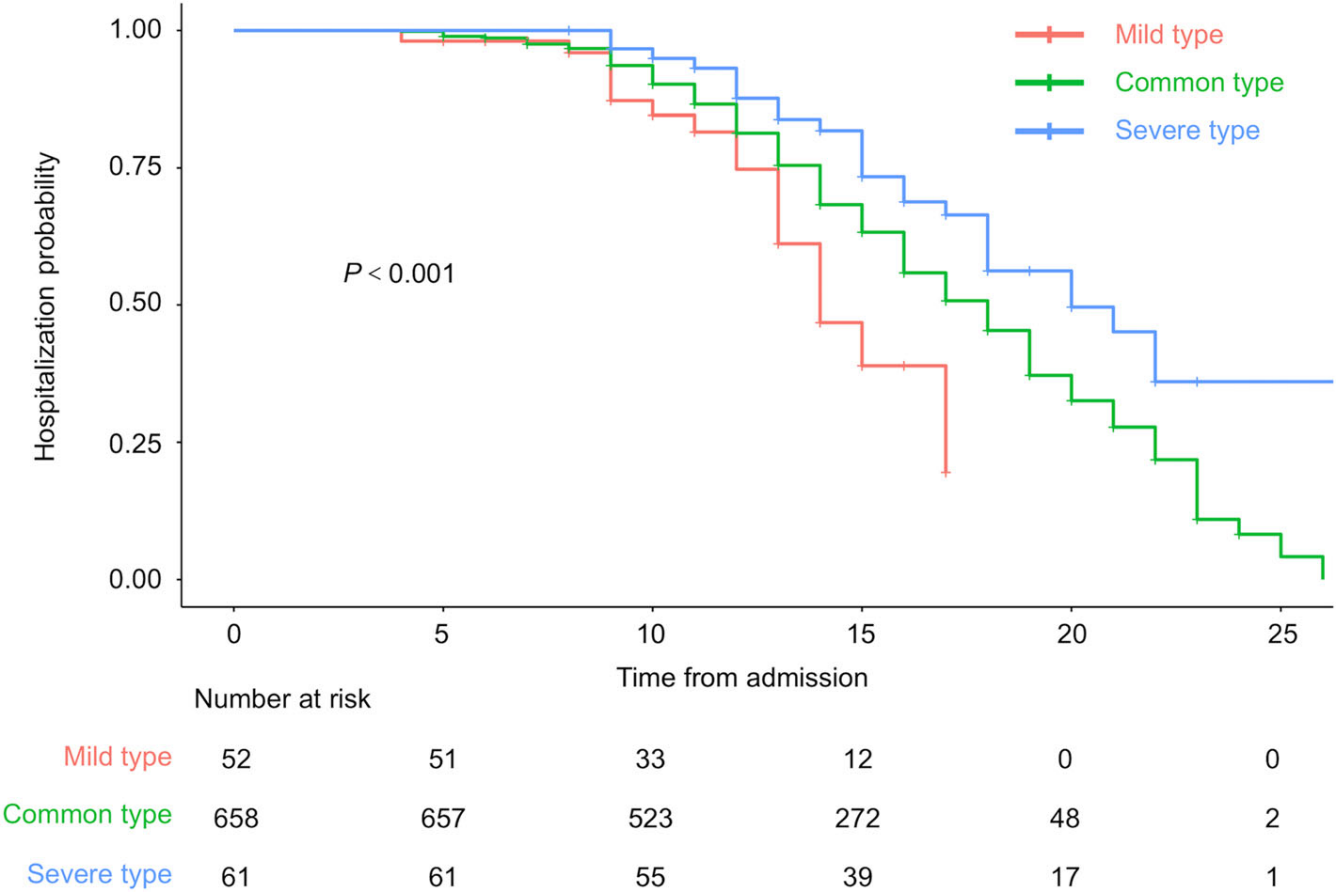
Kaplan–Meier analysis showed a significant difference in hospitalization time among mild type, common type and severe type

At the endpoint of our study, 21 cases (40.4%) of mild type, 273 cases (41.5%) of common type, 27 cases (44.3%) of severe type, and 1 case (5.9%) of critical type discharged from hospital, and none of the patients died. Kaplan-Meier analysis showed a significant difference in hospitalization time among mild type, common type and severe type (Fig. 1).

### Risk factors for the severity of illness in patients with COVID-19

As shown in Table 4, the variables with *P* <  0.05 in the univariate ordinal logistic regression model were selected into the multivariate ordinal logistic regression model for the severity of illness in patients with COVID-19. There was no multicollinearity within the variables in the final model. Results of multivariate ordinal logistic regression showed the severity of illness was relevant to the age-grading, sex, fever, cough, hemoptysis, gastrointestinal symptoms and hypertension. Multivariate ordinal logistic regression demonstrated increasing odds of the severity of illness in patients with COVID-19 associated with male (odds ratio [*OR*] = 1.7, 95% confidence interval [*CI*]: 1.2–2.6, *P* = 0.008), fever (*OR* = 3.6, 95% *CI*: 2.1–6.3, *P* < 0.001), cough (*OR* = 1.7, 95% *CI*: 1.0–2.9, *P* = 0.041), hemoptysis (*OR* = 3.4, 95% *CI*: 1.1–10.3, *P* = 0.032), gastrointestinal symptoms (*OR* = 1.9, 95% *CI*: 1.0–3.5, *P* = 0.047), hypertension (*OR* = 2.6, 95% *CI*: 1.2–5.6, *P* = 0.013). With the increase of age-grading, the risk for the severity was gradually higher (≤ 18 years [*OR* = 1.0], 19–40 years [*OR* = 12.7, 95% *CI*: 4.5–36.0, *P* <  0.001], 41–65 years [*OR* = 14.8, 95% *CI*: 5.2–42.1, *P* <  0.001], ≥ 66 years [*OR* = 56.5, 95% *CI*: 17.1–186.5, *P* < 0.001]).

**Table 4:** Risk factors for the severity of illness in patients with COVID-19

**Variables**	**Univariate analysis**			**Multivariate analysis**		
	***OR***	**95% *CI***	***P*value**	***OR***	**95% *CI***	***P*value**
Age (years)						
≥ 66	111.8	38.7–323.2	**< 0.001**	56.5	17.1–186.5	**< 0.001**
41–65	29.0	11.2–74.9	**< 0.001**	14.8	5.2–42.1	**< 0.001**
19–40	16.4	6.3–42.3	**< 0.001**	12.7	4.5–35.9	**< 0.001**
≤ 18	1.0			1.0		
Sex (male)	1.6	1.1–2.3	**0.024**	1.7	1.2–2.6	**0.008**
Current smoker	1.5	0.7–3.1	0.255			
Family cluster	0.5	0.3–0.8	**0.007**	0.9	0.5–1.4	0.579
Exposure History in Wuhan	1.1	0.8–1.6	0.608			
Time from illness onset to first hospital admission	1.1	1.0–1.2	**< 0.001**	1.1	1.0–1.1	0.071
**Symptoms**						
Fever	4.3	2.6–7.1	**< 0.001**	3.6	2.1–6.3	**< 0.001**
Cough	2.7	1.8–4.1	**< 0.001**	1.7	1.0–3.0	**0.041**
Sputum production	2.2	1.5–3.4	**< 0.001**	1.3	1.0–2.1	0.366
Hemoptysis	7.7	2.8–21.3	**< 0.001**	3.4	1.1–10.3	**0.032**
Sore throat	0.8	0.4–1.3	0.345			
Nasal obstruction	0.3	0.2–0.7	**0.005**	0.6	0.2–1.4	0.223
Myalgia	2.6	1.5–4.5	**0.001**	1.8	1.0–3.4	0.063
Fatigue	1.8	1.1–2.8	**0.022**	1.3	0.7–2.2	0.371
gastrointestinal symptoms	2.1	1.2–3.6	**0.012**	1.9	1.0–3.5	**0.047**
Headache	2.0	1.1–3.6	**0.030**	2.0	1.0–4.0	0.052
**Coexisting disorder**						
Any	3.8	2.5–5.9	**< 0.001**	1.2	0.6–2.4	0.660
Hypertension	4.9	3.0–7.8	**< 0.001**	2.6	1.2–5.6	**0.013**
Heart disease	2.5	0.6–10.1	0.189			
Diabetes	2.4	1.2–4.6	**0.009**	0.8	0.4–1.9	0.675
Chronic obstructive pulmonary disease	42.0	4.9–363.9	**0.001**	7.7	0.8–75.6	0.081
Cancer	7.3	1.5–35.2	**0.013**	3.7	0.6–22.2	0.149
Chronic liver disease	1.6	0.6–3.9	0.335			
Chronic renal disease	4.3	0.9–20.6	0.065			

*OR* Odds ratio, *CI* Confidence interval

## Discussion

The outbreak of COVID-19 is now becoming an enormous threat to public health. With further research of the structure and infection mechanism of respiratory, scientists found angiotensin converting enzyme 2 (ACE2) might be the site of SARS-CoV-2 binding on the surface of cells, with the same route of infection of SARS-CoV [14]. It has been proved that ACE2 might play an important role in virus transmission and infection. SARS-CoV-2 not only attacked the lung but also caused damages to many other organs, including heart, kidney, liver and central nervous system [3, 6, 15, 16]. The diagnosis of COVID-19 was complicated by the diversity in symptoms, imaging findings and the severity of illness, therefore, describing features of each subtype and exploring risk factors for the severity of illness could help clinicians to better tackle with this disease.

In this respective study, the median age of critical types was higher than the other three types, with the reason of low immunity and degeneration of related physiological function in older. The length of the incubation period is related to many factors, such as the number of pathogens, the time required for toxin production and transmission, and human immunity. The incubation period was shorter in severe type, probably due to poor immunity and higher viral load [17]. Fever and cough were the dominant symptoms in all subtypes, while hemoptysis was rare. Fever is a protective mechanism by activating the immune systems to resist pathogens, and cough is a reflective defense against invaders. When an individual was infected, the above two symptoms generally appeared at early stage. Although hemoptysis was an atypical symptom, it was reported that there was COVID-19 patient admitted only with hemoptysis as the initial symptom [18]. Moreover, we should be alert to the patients who didn’t have a fever, due to 6.6% of severe type without fever in our study. Hypertension and diabetes were the most pervasive underlying diseases in all subtypes, and the proportion of them were higher in critical type. In consideration of the aged constituting the majority of the critical type, it is common that the rates of comorbidities increased.

According to the laboratory results, the decrease of lymphocyte occurred in 70.6% of critical type. The decrease of lymphocyte was a prominent feature of critical type in our cohort which was consistent with a previous study [19]. SARS-CoV-2 might mainly act on lymphocyte, especially T lymphocytes, as did SARS-CoV [6]. In addition, the increased AST and LDH implied a potential of liver and heart damage which was more common in severe and critical types.

Currently, there is no effective antiviral treatment for COVID-19 [20]. In the light of the previous clinical experience, interferon-α, lopinavir/ritonavir and arbidol were applied for antiviral therapy in our hospital, however, the therapeutic regimen was not researched a consensus among hospitals. A retrospective study identified that proper use of corticosteroid in critical type with SARS could lead to a lower mortality and shorter hospitalization stay [21]. In our study, the dosage of glucocorticoids was limited to 40–80 mg/d to avoid side effects. Until 12 February 2020, 21 cases (40.4%) of mild type, 273 cases (41.5%) of common type, 27 cases (44.3%) of severe type, and 1 case (5.9%) of critical type discharged from hospital.

Compared with initial patients infected with SARS-CoV-2 in Wuhan, the illness condition of patients in Zhejiang Province are relatively milder. None of the patients died at the end of our follow-up. This feature is obviously different from researches in Wuhan which reported a higher mortality [10, 19]. At early stage of outbreak of COVID-19 in Wuhan, shortage of local medical resources, insufficient understanding of this disease and no effective drugs might contribute to this phenomenon.

Several risk factors for the severity of illness in patients with COVID-19 were identified in our study including male, fever, cough, hemoptysis, gastrointestinal symptoms, hypertension, and higher age-grading. Several studies demonstrated that differences in COVID-19 disease prevalence and severity were associated with sex, which was similar to our results [3, 6, 19]. One study, using single-cell sequencing, found that expression of ACE2 was more predominant in Asian men [22]. During the evolution, females develop enhanced innate and adaptive immune responses than males and are less susceptible to viral infections [23]. These above two points might be the reasons for the higher prevalence and severity of COVID-19 in men than in women. Additionally, in our study, the presence of any one of the comorbidities was higher in male compared with female (30.7% vs 24.4%, *P* = 0.048). We thought this result might also partly explain why men are more prone to severity illness.

Our study found increasing odds of the severity of illness was associated with gastrointestinal symptoms. A bioinformatics analysis on single-cell transcriptomes demonstrated that ACE2 was not only highly expressed in the lung AT2 cells, esophagus upper and stratified epithelial cells but also in absorptive enterocytes from ileum and colon. Recently, two independent laboratories from China declared that they have successfully isolated live SARS-CoV-2 from the stool of patients [24]. An increasing number of studies remind us that digestive system might serve as an alternative route of infection for SARS-CoV-2 [25]. We consider the digestive tract transmission of SARS-CoV-2 might impair the function of intestinal mucosal barriers and increase the production of inflammatory factors, further aggravating the severity of illness.

In addition, COVID-19 patients combined with hypertension were at higher risk for the illness severity in our study. Renin-angiotensin system (RAS) plays an important role in the pathogenesis of hypertension. It could be simply summarized as two axes, one is ACE-Ang II-AT1 axis responsible for constriction of blood vessels, and the other is ACE2-Ang-(1–7)-Mas axis with the opposite effect [26, 27]. Generally, the two axes could interact with each other and maintain the blood pressure balance. However, the balance would be broken in hypertension, with the result of a lower expression of ACE2. Once infected by SARS-CoV-2, the level of ACE2 would be even lower, followed by intensified Ang II activity. Ang II would further promote vasoconstriction, increase vascular permeability and mediate inflammatory responses, leading to illness aggravation.

There are several limitations in our study. Firstly, the retrospective design of our study might affect integrality of data and diminish its credibility, and more prospective cohort studies should be on the agenda in the future. Secondly, patients enrolled in our study only come from Zhejiang Province, and large-scale researches at the national level were urgently needed which could provide more reliable and comprehensive data. Thirdly, changes of the illness in different subtypes needed to be further investigated. A model for predicting the changes of disease was necessary for clinicians to better guide treatments. Moreover, laboratory results were not included in ordinal logistic regression model to explore risk factors for the severity of illness, due to the normal range of some indicators varied from different hospitals.

## Conclusions

In summary, our study reported the largest cases of patients with COVID-19 in Zhejiang Province, and indicated risk factors of illness severity which was of great significance for early identification and prompt treatment. Based on the research findings, we recommend that clinicians should pay close attention to these features in patients with COVID-19 including older age, male, fever, cough, hemoptysis, gastrointestinal symptoms and hypertension, and strengthen self-protection during the outbreak of COVID-19.

## Data Availability

All datasets are presented in the main paper.

## Abbreviations

WHO: World Health Organization
SARS-CoV-2: Severe acute respiratory syndrome coronavirus 2
SARS-CoV: Severe acute respiratory syndrome coronavirus
MERS-CoV: Middle East respiratory syndrome coronavirus
COVID-19: Coronavirus disease 2019
RT-PCR: Real-time reverse transcriptase polymerase chain reaction
China CDC: Chinese Center for Disease Control and Prevention
ALB: Albumin
AST: Aspartate aminotransferase
BUN: Blood urea nitrogen
CR: Creatinine
CK: Creatinine kinase
LDH: Lactate dehydrogenase
CRP: C-reactive protein
PCT: Procalcitonin
ALT: Alanine aminotransferase
TB: Total bilirubin
INR: International normalized ration
ECMO: Extracorporeal membrane oxygenation
CRRT: Continuous renal-replacement therapy
RAS: Renin-angiotensin system
ACE2: Angiotensin converting enzyme 2
ACE: Angiotensin converting enzyme
Ang II: Angiotensin II
AT1: Angiotensin type 1
Ang-(1–7): Angiotensin-(1–7)
